# The association of early life stress with IQ‐achievement discrepancy in children: A population‐based study

**DOI:** 10.1111/cdev.13825

**Published:** 2022-07-13

**Authors:** Isabel K. Schuurmans, Annemarie I. Luik, Donna A. de Maat, Manon H. J. Hillegers, M. Arfan Ikram, Charlotte A. M. Cecil

**Affiliations:** ^1^ Department of Epidemiology Erasmus MC, University Medical Center Rotterdam Rotterdam The Netherlands; ^2^ The Generation R Study Group Erasmus MC, University Medical Center Rotterdam Rotterdam The Netherlands; ^3^ Department of Child and Adolescent Psychiatry/Psychology Erasmus MC, University Medical Center Rotterdam Rotterdam The Netherlands; ^4^ Department of Psychology, Education, and Child Studies Erasmus School of Social and Behavioural Sciences, Erasmus University Rotterdam Rotterdam The Netherlands; ^5^ Department of Biomedical Data Sciences Molecular Epidemiology, Leiden University Medical Center Leiden The Netherlands

## Abstract

Early life stress (ELS) is associated with lower IQ and academic achievement; however, it remains unclear whether it additionally explains their discrepancy. In 2,401 children (54% girls, 30.2% migration background) from the population‐based study Generation R Study, latent factors of prenatal and postnatal (age 0–10) ELS were estimated, and IQ‐achievement discrepancy (age 12) was quantified as variance in academic achievement not explained by IQ. ELS was prospectively associated with larger IQ‐achievement discrepancy (*β*_prenatal_ = −0.24; *β*_postnatal_ = −0.28), lower IQ (*β*_prenatal_ = −0.20; *β*_postnatal_ = −0.22), and lower academic achievement (*β*_prenatal_ = −0.31; *β*_postnatal_ = −0.36). Associations were stronger for latent ELS than for specific ELS domains. Results point to ELS as a potential prevention target to improve academic potential.

Intelligence quotient (IQ) facilitates knowledge acquisition and is associated with greater academic achievement for most children (Borghans et al., 2016). However, some children show a discrepancy between their IQ and academic achievement (Borghans et al., 2016; Reis & McCoach, 2000), that is, their school performance differs relative to what would be expected based on intelligence. This IQ‐achievement discrepancy could either manifest as higher or lower academic achievement than expected. The latter can be considered particularly detrimental, as it indicates that children are not meeting their full potential, sometimes referred to as academic underachievement. Understanding what factors drive the IQ‐achievement discrepancy is important, as lower academic achievement than expected based on IQ has been shown to hamper self‐actualization; it is associated with a range of adverse long‐term consequences, including delinquent behavior, social problems, lower quality of life, and poor employment prospects (Hoffmann, 2020; McCall et al., 1992). However, except for some evidence suggesting executive function might be particularly important in the IQ‐achievement discrepancy (Alloway & Alloway, 2010; Ng & Hodges, 2020), little is known about what other factors underlie the IQ‐achievement discrepancy.

Early life stress (ELS) describes an individual's exposure to single or multiple adverse events in prenatal life and childhood, leading to prolonged phases of stress (Lupien et al., 2009; Pechtel & Pizzagalli, 2011). Robust evidence shows that exposure to prenatal (i.e., the fetal period) and postnatal (i.e., from birth onwards) ELS is harmful and predicts poor developmental outcomes on a social, emotional, and behavioral level (de Maat et al., 2021; Kingston et al., 2012; Liming & Grube, 2018). A number of individual prenatal and postnatal stressors have been linked to academic achievement and IQ separately (e.g., poverty and childhood maltreatment; Kaya et al., 2016; Young‐Southward et al., 2020). A few studies have, however, used more comprehensive measures of ELS. These have shown that cumulative stressors experienced in the context of school, family, and neighborhood negatively relate to academic achievement at school age (Morales & Guerra, 2006), that deprivation is associated with executive functioning at school age (Vogel et al., 2021), and that prenatal early life stress is associated with lower IQ at age 6 (Cortes Hidalgo et al., 2020).

Despite growing evidence on the association of ELS with academic achievement and IQ, there are three important knowledge gaps. First, while ELS has been examined in relation to IQ and academic achievement separately, it remains unclear whether ELS further explains the discrepancy between these outcomes. Second, although both prenatal and postnatal ELS have been shown to be negatively associated with cognitive outcomes and academic performance separately (Cortes Hidalgo et al., 2020; Pechtel & Pizzagalli, 2011), these periods have not been examined together in one population. It is of interest to address the role of timing, given the evidence of continuity in ELS over time (Dipietro et al., 2008; Najman et al., 2010). Third, despite robust evidence that risks co‐occur (Ackerman et al., 1999; Appleyard et al., 2005; Evans, 2004), research to date has primarily focused on individual stressors in isolation (Kaya et al., 2016; Morales & Guerra, 2006; Vogel et al., 2021; Young‐Southward et al., 2020). As such, it remains unclear whether the reported associations with IQ and academic achievement are specific to individual ELS domains or shared between them. Overall, understanding the role of timing and specificity of ELS effects on IQ‐achievement discrepancy could lead to improved risk assessment and more targeted prevention strategies.

To address these knowledge gaps, the present study examined whether exposure to co‐occurring stressors in early life is related to the IQ‐achievement discrepancy in a general population of children followed from pregnancy up to 13 years old. With this study, we shed light on (i) the association of ELS (i.e., prenatal and postnatal up to age 10) with the IQ‐achievement discrepancy, (ii) whether associations vary depending on the timing of ELS, and (iii) the relative importance of specific ELS domains and the shared variance across them in driving associations with the IQ‐achievement discrepancy. Our analyses of the association between ELS and IQ and academic achievement can be considered to be confirmatory, given the expected negative association between ELS and these outcomes. Our analysis of the association between ELS and IQ‐achievement discrepancy can be considered to be exploratory, as here we did not specify any initial hypotheses.

## METHOD

### Participants

Information from children and their caregivers was obtained from The Generation R Study, a population‐based prospective cohort from fetal life onwards (Kooijman et al., 2017). In short, pregnant mothers within Rotterdam, The Netherlands, were eligible to enroll in The Generation R Study if they had a delivery date between April 2002 and January 2006. In total, 9,778 mothers were enrolled. The study sample is largely representative of the underlying population, although included participants are more likely to have a higher educational level and income. These mothers, their partners, and their children took part in a diverse array of assessments, including behavioral, cognitive, and sociodemographic measures.

A flowchart of the study population is provided in Supplemental Figure S1. Children were included in this study if they participated in a research center visit around age 13 (*n* = 4,929). Participants were excluded if their prenatal (*n* = 859) or postnatal (*n* = 281) ELS scores could not be computed due to high frequencies of missing ELS items (more than 50% of the ELS items per period), when not all four IQ tests were assessed around age 13 (*n* = 168), or when academic achievement around age 12 (*n* = 1,220) was not available. The final sample included 2,401 children. The characteristics of the study sample are shown in Table 1

**TABLE 1:** Characteristics of The Generation R Study population

	Mean	*SD*	*n*	%
Child characteristics				
Sex				
Boy			1,105	46.0
Girl			1,296	54.0
Age child at academic assessment (years)	11.88	0.46		
Age child at IQ assessment (years)	13.58	0.30		
Mean age difference between assessments (years)	1.70	0.54		
Ethnicity				
Dutch			1,670	69.5
Non‐Dutch Western			214	8.9
Non‐Western			512	21.3
Missing			5	0.2
IQ	105.92	12.33		
Academic achievement^a^	0.00	1.00		
IQ‐achievement discrepancy^a^	0.00	1.00		

^a^ Both academic achievement and the IQ‐achievement discrepancy are standardized.

The general design, research aims, and specific measurements of The Generation R Study have been approved by the Medical Ethical Committee of Erasmus MC, in accordance with the Declaration of Helsinki of the World Medical Association. Written informed consent was obtained from the parents on behalf of the child. For data collected when the child was 12 years or older, the child also provided written informed consent.

### Early life stress

Two ELS scores were created, one for the prenatal period, including items measured during pregnancy, and one for the postnatal period, including items measured from birth up to 10 years of age. In line with previous work (Cecil et al., 2014, Cortes‐Hidalgo et al., 2020), single ELS items (coded as yes or no) were combined into five individual ELS domains: (i) life events (e.g., death in the family), (ii) contextual stress (e.g., poor housing conditions, financial difficulties), (iii) parental stress (e.g., parental psychopathology, early parenthood), (iv) interpersonal stress (e.g., family relationship difficulties), and (v) direct victimization of the child (e.g., the child is bullied or physically hurt by someone—only available postnatally). Items were derived from a broad range of self‐report questionnaires filled in by primary caregivers, partners, and teachers (see Supplemental Tables S1 and S2 for details).

The missing value frequencies of variables within the individual ELS domains ranged between 0.2% and 32.2% (*M* = 7.3%; see Supplemental Tables S1 and S2). Only 54.1% of the participants had complete prenatal information and only 43.2% had complete postnatal information (see Supplemental Table S3 for proportion complete and incomplete cases per developmental period). Furthermore, fully complete cases had more favorable health and socioeconomic status (Supplemental Table S4). Therefore, missing ELS items were imputed by Multivariate Imputation by Chained Equations (mice) (Van Buuren & Groothuis‐Oudshoorn, 2011) using 30 imputed datasets and 60 iterations. Our imputation strategy was based on Van Buuren (2018). Domains were passively imputed, meaning that for each completed imputed dataset, domain scores were computed by summing its ELS items and dividing this by the total number of items within that domain. The following information was used to impute the missing ELS items: (i) ELS items (specific to the domain), (ii) domain sum scores, (iii) the IQ‐achievement discrepancy, and (iv) auxiliary variables (Van Buuren, 2018), including variables related to the mother (age, BMI, marital status, low education, smoking, parity), parent (depressive symptoms measured with Brief Symptom Inventory Derogatis & Melisaratos, 1983), and child (birth weight, ethnicity, sex). The algorithm converged well (see Supplemental Figure S2 for trace plots for 6 example ELS items). Furthermore, the distribution of imputed versus observed values showed higher risk in those with missing data (see Supplemental Figure S3 for density plots for 6 example ELS items).

**FIGURE 1:**
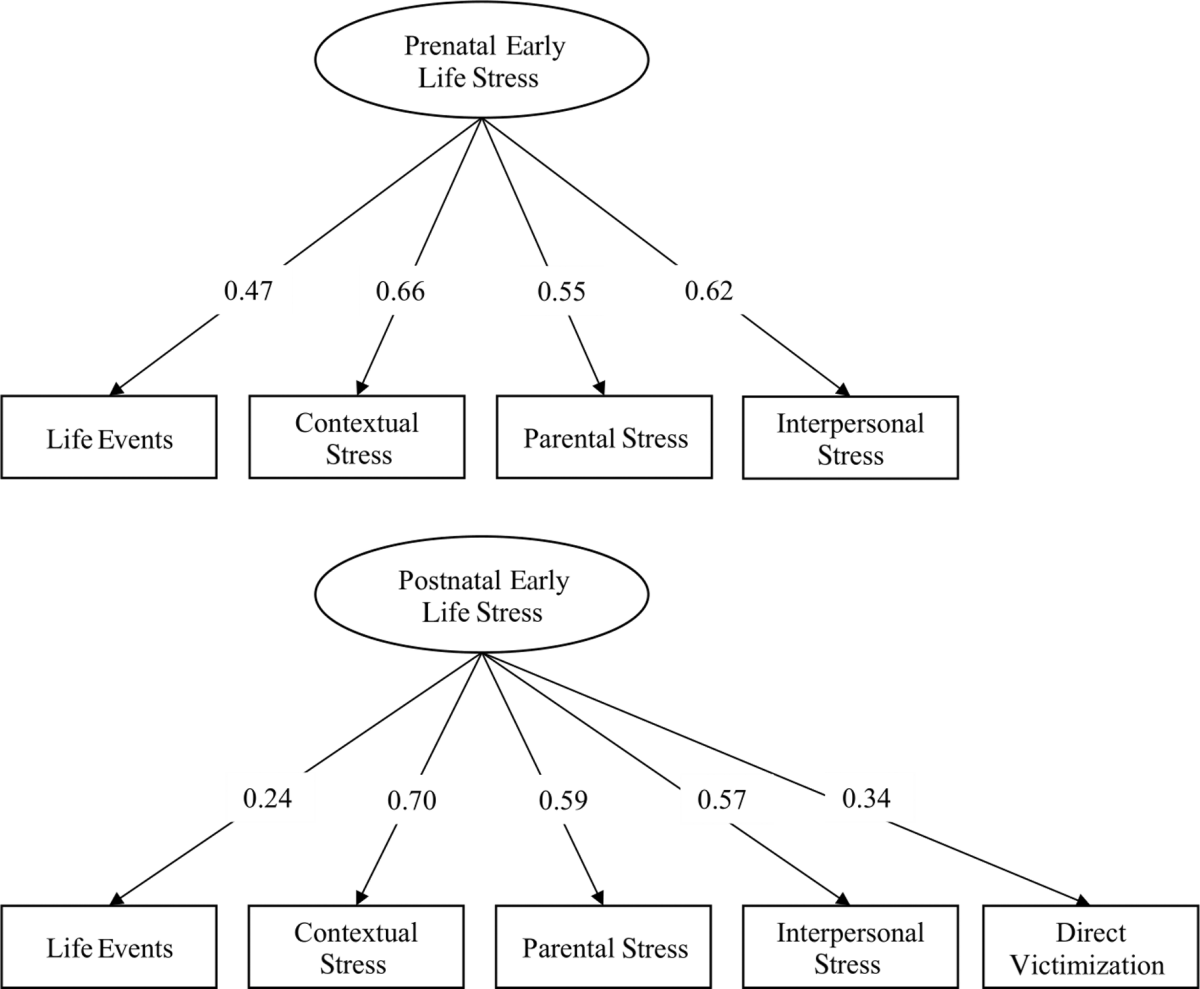
Factor loadings of the prenatal and postnatal early life stress score. All factor loadings are standardized. Upper panel depicts prenatal ELS score. Lower panel depicts the postnatal ELS score.

Based on the individual ELS domains, we estimated a latent prenatal and postnatal ELS score, using structural equation modeling performed with the Lavaan package version 0.6‐9 (Rosseel, 2012) in R version 3.6.3 (R Core Team, 2020). The ELS score for each developmental period was defined as a reflective latent variable, using the different domains as indicators. The score reflects what is common to the different individual ELS domains (i.e., shared variance) with higher scores implying more ELS. Factor loadings for all ELS domains can be found in Figure 1. The latent variable model showed a good model fit for both the prenatal and postnatal period (Iacobucci, 2010): prenatal model: chi‐square statistics (*χ* ^2^) (2) = 7.42, *p* = .024, standardized root mean square residual (SRMR) = .013, and comparative fit index (CFI) = .995. Postnatal model: *χ* ^2^ (5) = 27.12, *p* < .001, SRMR = .027, and CFI = .978.

### IQ‐achievement discrepancy

#### Intelligence quotient

To determine the IQ‐achievement discrepancy, we measured IQ and academic achievement. For IQ, we used the Wechsler Intelligence Scale for Children‐Fifth Edition (WISC‐V) (Wechsler, 2014). Similarly to what has been done with previous versions of the WISC in other cohorts (Horwood et al., 2008), we assessed only a subset of the WISC‐V. We used Matrix reasoning to measure fluid reasoning, Digit Span for working memory, Coding for processing speed, and Vocabulary for verbal comprehension. The WISC‐V was administered by a trained examiner during the research center visit when children had a mean age of 13.6 (*SD* = 0.3). Digit Span and Vocabulary were administered verbally. Coding and Matrix Reasoning were administered digitally, but a small portion was acquired through the paper‐pencil version. The scores on the subtests were scaled and summed, and this sum score was converted to IQ using a conversion table that was created by Pearson (Blok et al., 2022). Pearson has assessed the reliability of the abbreviated version of the WISC‐V and found it to have a Pearson correlation of .93 across age 6—16 with the full‐scale IQ in other samples that used the WISC‐V (Blok et al., 2022).

#### Academic achievement

Academic achievement was examined using a national standardized school achievement test recorded by the Dutch Central Institute for Test Development (Cito) (Van Boxtel et al., 2010), which is administered in the final year of primary education in the Netherlands. The mean age during academic assessment was 11.9 (*SD* = 0.5). The test is used by 85% of the Dutch primary schools (Van der Lubbe, 2007) and consists of 160 multiple choice questions divided into two domains: 60 questions assessing arithmetic and 100 questions assessing language ability. Together, the questions result in a standardized score ranging between 501 and 550. Each school year, the questions are revised and changed. Therefore, raw subscale scores are not comparable between different test years, while the standardized scores are. The reliability of the Cito test is high (Cronbach's alpha > .90; Van Boxtel et al., 2010). Scores were collected through the teachers of participating children (*n* = 1,820) or maternal self‐report (*n* = 581). Previous work of our group has shown an adequate validity of mother‐reported Cito test scores (Steenkamp et al., 2021).

#### IQ‐achievement discrepancy computation

The IQ‐achievement discrepancy was quantified using a regression method, based upon Lau and Chan (2001). A regression model was performed with IQ as a predictor and academic achievement as an outcome. We saved the standardized residuals of the model, which indicate the variance in academic achievement not explained by IQ. These standardized residuals are then used as a continuous measure of the IQ‐achievement discrepancy. Negative values indicate lower academic achievement than expected, whereas positive values indicate higher academic achievement than expected.

### Other variables

Age of the child was obtained during academic assessment. Sex of the child was obtained from midwives and hospital registries. Ethnicity of the child was obtained by self‐report during pregnancy, being categorized according to the classification of Statistics Netherlands (2004), which distinguishes “Western” (European, North‐American, and Oceanian) and “non‐Western” (Turkish, Moroccan, Indonesian, Cape Verdean, Surinamese, and Antillean).

### Statistical analyses

Statistical analyses were performed using R version 3.6.3 (R Core Team, 2020). Before running analyses, assumptions for statistical tests were checked and results were only reported if the assumptions were met. As a first step, we performed a non‐response analysis comparing the included participants with the follow‐up dataset at age 13, and with the baseline sample.

For our main analyses, we examined associations between the latent ELS scores and IQ‐achievement discrepancy using linear regression models. Separate models were run for prenatal and postnatal ELS as independent predictors. Due to the high correlation between prenatal and postnatal ELS, simultaneous examination of both periods as predictors was not possible. To further understand the differences between underachievers and overachievers, we stratified the IQ‐achievement discrepancy into three categories: underachieving (> 1 *SD* below the mean), normal achieving (within 1 *SD* of the mean) and, overachieving children (> 1 *SD* above the mean). The association of ELS with the achievement categories was assessed with multinomial logistic regression, with normal achieving as the reference category. To maximize comparability with previous studies in the field, we also examined the separate associations of ELS with academic achievement and IQ.

Additionally, we analyzed the individual ELS domains in relation to the IQ‐achievement discrepancy. We assessed the association in individual models as well as in a simultaneous model to examine the unique contribution of each ELS domain, over and above the others. The separate associations of the individual ELS domains with IQ and academic achievement were also examined.

Analyses were adjusted for number of tests performed (the number of individual regression models per outcome, being 13 tests), using the Benjamini–Hochberg false discovery rate correction (Benjamini & Hochberg, 1995). All analyses were adjusted for sex and age of the child during academic assessment. Given the low but significant point‐biserial correlation between sex and the IQ‐achievement discrepancy, *r* = −0.08, *p* < .001, we investigated sex moderation as a post hoc analysis. Furthermore, we performed three sensitivity analyses. First, the association between ELS and verbal IQ‐measures might be confounded by ethnicity. Therefore, a sensitivity analysis was performed, which repeated all analyses in a subpopulation consisting only of Dutch participants. Second, to assess whether associations hold when more stringent exclusion criteria are used, we have repeated our analyses in a subpopulation that excluded participants with 25% or more missing data in the prenatal or postnatal ELS measure. Third, to assess sample selection effects, we have repeated our analyses by not excluding those participants with missing information on either academic achievement or IQ (*n* = 4,126). In this sensitivity analyses, missing IQ and academic achievement were imputed using Multivariate Imputation by Chained Equations (mice) (Van Buuren & Groothuis‐Oudshoorn, 2011). Further details on the imputation strategy are provided in the legend of Supplemental Table S10.

## RESULTS

### Descriptive statistics and correlations

The characteristics of the study sample are shown in Table 1. A non‐response analysis can be found in Supplemental Table S5. Individual ELS domains were weakly to moderately inter‐correlated within and between developmental periods (Supplemental Table S6). Within our structural equation model, latent prenatal ELS strongly correlated with latent postnatal ELS, *r* = .94, *p* < .001. Of the 44 individual risk factors assessed in the prenatal period, participants had on average 4.04 risk factors (*SD* = 3.54). Of the 51 risk factors assessed in the postnatal period, participants had on average 5.78 risk factors (*SD* = 3.44).

### Prenatal and postnatal early life stress

**FIGURE 2:**
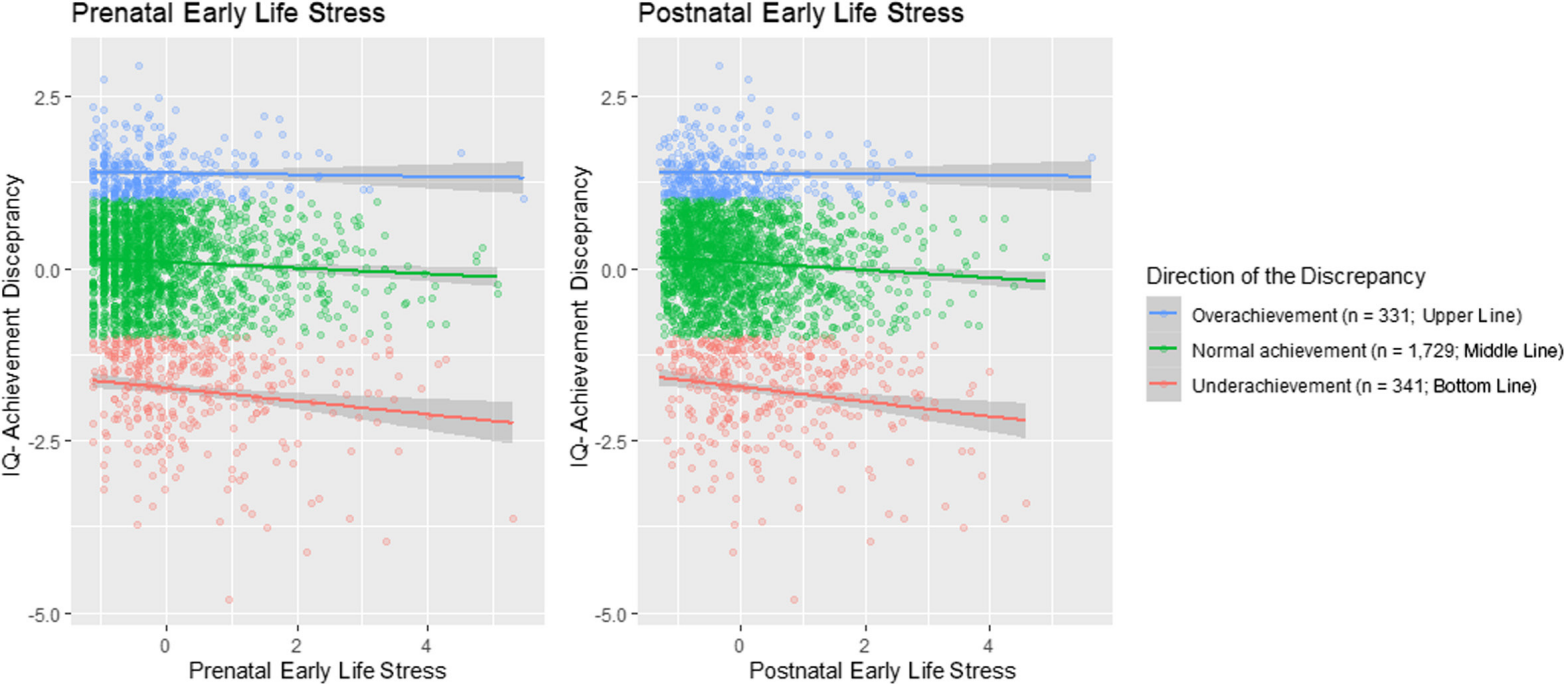
The relation between early life stress and IQ‐achievement discrepancy within different achievement categories (i.e., overachievement, normal achievement and underachievement). The figure illustrates the relation between early life stress and the IQ‐achievement discrepancy. Lines are stratified on the outcome variable, meaning different lines belong to different achievement categories. The blue (upper) line represents children who overachieve (academic achievement >1 *SD* higher than what would be expected based on IQ), the green (middle) line show those who achieve as would be expected based on their IQ, and the red (bottom) line represents children who underachieve (academic achievement >1 *SD* lower than what would be expected based on IQ). This figure shows that higher levels of ELS are associated with a greater IQ‐achievement discrepancy, and that this association is linear (i.e., ELS is associated with lower, not higher, academic achievement than expected based on IQ; this is inferred from the direction of the slope, that does not have opposite directions for underachievement and overachievement).

Prenatal and postnatal ELS were individually associated with lower academic achievement than would be expected based on IQ (Table 2). When prenatal ELS increased with 1 *SD* (equaling 3.54 risk factors), expected academic achievement decreased with 0.24 *SD*. An increase of 1 *SD* in postnatal ELS (equaling 3.44 risk factors) corresponded with a decrease of 0.28 in expected academic achievement. The association between ELS and the IQ‐achievement discrepancy was linear. As such, compared to normal achievement, higher ELS was associated with higher odds for underachieving, *B*_prenatal_ = 0.48, *p* < .001, OR = 1.61; *B*_postnatal_ = 0.61, *p* < .001, OR = 1.85, as well as lower odds for overachieving, *B*_prenatal_ = −0.22, *p* = .009, OR = .81; *B*_postnatal_ = −0.20, *p* = .015, OR = .82. Figure 2 illustrates the relation between ELS and IQ‐achievement discrepancy for underachievers, normal achievers and overachievers. Both prenatal and postnatal ELS were related to lower IQ and academic achievement in the individual models (Table 3).

**TABLE 2:** The association between early life stress for both the prenatal and postnatal period and IQ‐achievement discrepancy (individual regression models)

	IQ‐achievement discrepancy			
	*Β*	95% CI	*p*‐value	Adjusted *p*
Prenatal early life stress	−0.24	−0.29, −0.18	<.001	<.001
Life events	−0.06	−0.10, −0.01	.008	.104
Contextual stress	−0.14	−0.18, −0.10	<.001	<.001
Parental stress	−0.21	−0.25, −0.17	<.001	<.001
Interpersonal stress	−0.06	−0.10, −0.03	.001	.013
Postnatal early life stress	−0.28	−0.34, −0.22	<.001	<.001
Life events	−0.06	−0.10, −0.02	.007	.095
Contextual stress	−0.17	−0.21, −0.13	<.001	<.001
Parental stress	−0.12	−0.16, −0.08	<.001	<.001
Interpersonal stress	−0.24	−0.28, −0.20	<.001	<.001
Direct victimization	−0.07	−0.12, −0.02	.003	.040

*Note*: Negative IQ‐achievement discrepancy value represents lower actual achievement than would be expected based on IQ. Each row corresponds to one model output. Regression estimates are standardized. All models are adjusted for sex and age at academic assessment. We adjusted *p*‐values for 13 tests performed, using the Benjamini–Hochberg false discovery rate correction.

**TABLE 3:** The association between early life stress for both the prenatal and postnatal period and IQ and academic achievement (individual regression models)

	Intelligence quotient				Academic achievement			
	*B*	95% CI	*p*‐value	Adjusted *p*	*B*	95% CI	*p*‐value	Adjusted *p*
Prenatal early life stress	−0.20	−0.25, −0.16	<.001	<.001	−0.31	−0.37, −0.25	<.001	<.001
Life events	−0.04	−0.08, −0.00	.031	.400	−0.07	−0.11, −0.03	<.001	.011
Contextual stress	−0.11	−0.14, −0.07	<.001	<.001	−0.18	−0.22, −0.14	<.001	<.001
Parental stress	−0.20	−0.24, −0.16	<.001	<.001	−0.29	−0.33, −0.25	<.001	<.001
Interpersonal stress	−0.16	−0.20, −0.13	<.001	<.001	−0.13	−0.17, −0.09	<.001	<.001
Postnatal early life stress	−0.22	−0.27, −0.17	<.001	<.001	−0.36	−0.42, −0.30	<.001	<.001
Life events	−0.07	−0.11, −0.03	<.001	.006	−0.09	−0.13, −0.05	<.001	<.001
Contextual stress	−0.13	−0.17, −0.09	<.001	<.001	−0.21	−0.25, −0.18	<.001	<.001
Parental stress	−0.06	−0.10, −0.03	.001	.013	−0.14	−0.18, −0.10	<.001	<.001
Interpersonal stress	−0.19	−0.23, −0.15	<.001	<.001	−0.31	−0.35, −0.27	<.001	<.001
Direct victimization	−0.10	−0.14, −0.06	<.001	<.001	−0.11	−0.16, −0.07	<.001	<.001

*Note*: Each row corresponds to one model output. Regression estimates are standardized. All models adjusted for sex and age at academic assessment. We adjusted *p*‐values for 13 tests performed, using the Benjamini–Hochberg false discovery rate correction.

### Individual ELS domains

The vast majority of individual ELS domains were significantly associated with a larger IQ‐achievement discrepancy, lower IQ, and lower academic achievement, as presented in Tables 2 and 3. When modeling all individual domains simultaneously as predictors, most domains were no longer significantly associated with outcomes. Only contextual and parental stress remained to be independently associated with all outcomes across developmental periods (see Table 4 for results on IQ‐achievement discrepancy, see Supplemental Table S7 for results on IQ and academic achievement).

**TABLE 4:** The association between early life stress for both the prenatal and postnatal period and IQ‐achievement discrepancy in simultaneous models

	IQ‐achievement discrepancy			
	*Β*	95% CI	*p*‐value	Adjusted *p*
Prenatal early life stress				
Life events	0.02	−0.03, 0.06	.462	1.000
Contextual stress	−0.06	−0.11, −0.02	.009	.117
Parental stress	−0.18	−0.22, −0.13	<.001	<.001
Interpersonal stress	−0.05	−0.09, 0.00	.044	.572
Postnatal early life stress				
Life events	−0.02	−0.06, 0.02	.289	1.000
Contextual stress	−0.07	−0.12, −0.03	.002	.004
Parental stress	−0.20	−0.24, −0.15	<.001	<.001
Interpersonal stress	−0.02	−0.07, 0.02	.347	1.000
Direct victimization	−0.01	−0.05, 0.04	.783	1.000

*Note*: Estimates are standardized. All models adjusted for sex and age at academic assessment. The first four rows correspond to one model output; the second five rows also correspond to one model output. Hence, two models were constructed: one for the prenatal and one for the postnatal period. The prenatal model comprised one regression model using as predictors: life events, contextual stress, parental stress, and interpersonal stress The postnatal model comprised one regression model using as predictors: life events, contextual stress, parental stress, interpersonal stress, and direct victimization. We adjusted *p*‐values for 13 tests performed, using the Benjamini–Hochberg false discovery rate correction.

### Sensitivity analyses

Findings were consistent when repeating the analyses in a subpopulation consisting of only Dutch participants (see Supplemental Table S8), in subpopulation with a maximum of 25% missing data (see Supplemental Table S9), and in a population in which missing information on outcomes was imputed (see Supplemental Table S10). As for potential sex moderation, while the association between prenatal ELS and the IQ‐achievement discrepancy was not moderated by sex, *B*_prenatal*sex_ = 0.10, *p* = .056; the association between *postnatal* ELS and the IQ‐achievement discrepancy showed marginally stronger associations for boys, *B*_postnatal*sex_ = 0.10, *p* = .040.

## DISCUSSION

This study aimed to characterize the role of ELS in the IQ‐achievement discrepancy, using prospective data from a population‐based study spanning pregnancy to late childhood. We highlight three main findings. First, we found that ELS was associated with a larger IQ‐achievement discrepancy, specifically lower achievement than expected based on IQ. Consistent with prior research, ELS was also individually associated with lower IQ and lower academic achievement in childhood. Second, we found that ELS from both developmental periods emerged as significant predictors of IQ‐achievement discrepancy (birth up to age 10). Third, overall ELS (modeled as a latent factor capturing shared variance between ELS domains) showed stronger associations with child outcomes than any individual ELS domains. Together, our findings point to prenatal and postnatal ELS as a significant predictor of IQ‐achievement discrepancy, with effects largely driven by the shared variance between ELS types.

The present findings demonstrate that ELS is prospectively associated with a larger IQ‐achievement discrepancy around age 12—a critical period of transition between middle and high school. Specifically, ELS is associated with lower academic achievement than expected based on IQ (i.e., underachievement), an important predictor of poor outcomes later in life (Hoffmann, 2020; McCall et al., 1992). Conversely, the odds of overachieving were lower when ELS was higher. According to the effect size guidelines for research in individual differences (Gignac & Szodorai, 2016), our effect sizes were typical to relatively large. This, and the finding that associations remained consistent across several sensitivity analyses, added confidence to our findings. We further confirm previous work by showing that ELS is also associated with both outcomes separately (Cortes Hidalgo et al., 2020; Pechtel & Pizzagalli, 2011). Of note, recent evidence suggests that the effects of ELS on cognitive outcomes can persist beyond childhood, associating, for example, with cognitive reserve later in life (Lesuis et al., 2018). Cognitive reserve refers to the individual differences in cognition that help to explain differential susceptibility to brain aging, pathology, or insult (Stern et al., 2020), therefore being considered an important construct for health later in life. Interestingly, cognitive reserve in adults has often been measured as the discrepancy between actual and predicted cognitive achievement (Stern et al., 2020), which parallels how the IQ‐achievement discrepancy is measured in children. Consequently, it could be possible that the association between ELS and cognitive reserve—indexed as the IQ‐achievement discrepancy—might emerge as early as in childhood. However, future research employing life course models will be needed to clarify the links between ELS, the IQ‐achievement discrepancy, and cognitive reserve across the lifespan. Overall, our results demonstrate that ELS is associated with a discrepancy between actual and predicted achievement later in development, although it is not yet clear how this discrepancy unfolds after childhood.

In the current study, we show that both prenatal and postnatal ELS are negatively associated with the IQ‐achievement discrepancy, and with IQ and academic achievement separately. Although the mechanisms behind prenatal and postnatal associations might be heterogeneous (Lupien et al., 2009), we found that ELS during both periods relates to a higher risk of poor cognitive and academic outcomes. Interestingly, we found that the effect of postnatal ELS was somewhat stronger than the effect of prenatal ELS. Potentially, prenatal ELS is associated with academic achievement indirectly, by predisposing the child to higher postnatal ELS. However, both periods comprise of items specific to the developmental timing of ELS, to which end prenatal and postnatal ELS are not fully comparable. Also, simultaneous examination of both prenatal and postnatal ELS as predictors within the same model is not possible, as a consequence of the high correlation between prenatal and postnatal ELS. The high correlation could potentially cause multicollinearity between the two predictors, which may lead to unstable coefficients (Vatcheva et al., 2016). Nonetheless, the finding that the correlation between prenatal and postnatal ELS was high indicates substantial continuity in ELS over time. This is in line with earlier work showing that exposure to adversity typically carries on from pregnancy to childhood (Dipietro et al., 2008; Najman et al., 2010). Of note, however, the association between ELS and the IQ‐achievement discrepancy may change over time, even within childhood. It will be important for future work to leverage repeated measure data to model ELS effects at different developmental stages.

Concerning the type of ELS examined, we found that global prenatal and postnatal ELS (measured as a latent score capturing shared variance across stressors) is associated with child IQ, academic achievement, and the IQ‐achievement discrepancy more strongly than any individual ELS domain. Indeed, although individual ELS domains were found to be associated with outcomes when examined separately, associations were substantially attenuated when these domains were modeled simultaneously, suggesting that these associations are mainly driven by shared variance across stressors. This is in line with previous research, showing that early life stressors co‐occur (Ackerman et al., 1999; Appleyard et al., 2005; Evans, 2004) and that the shared variance amongst these stressors is associated with poorer child functioning (Cortes Hidalgo et al., 2020; de Maat et al., 2021), following a dose‐response gradient. In addition, we extend this work by showing that the shared variance also is associated more strongly with the IQ‐achievement discrepancy. Together, these findings underscore the importance of comprehensively assessing stress exposures and accounting for their co‐occurence, as focusing on single risks may lead to overestimating the effects of any specific exposure. The interrelatedness of stress domains also illustrates how ELS may act as a cascade of events, in which stressors seem to follow up on each other, with potential cumulative effects. Future research is needed to delineate the mechanisms behind co‐occurrent stress and how to target this in intervention and prevention.

Our findings have two main implications. First, they suggest that early screening of ELS may help identify those at highest risk for academic underachievement, an important marker of later life outcomes, including delinquency, social problems, lower quality of life, and poor employment prospects (Hoffmann, 2020; McCall et al., 1992). More specifically, in children known to be exposed to ELS, teachers can be more vigilant to academic underachievement, monitor the IQ‐achievement discrepancy, and also offer support more quickly when adverse consequences present themselves. Second, our findings also indicate that academic underachievement is most strongly associated with the co‐occurrence of stressors, rather than any individual type of stressor. As such, the implementation of more comprehensive screening tools for ELS is warranted, as assessment of individual stressors in isolation is likely to underestimate effects on IQ‐achievement discrepancy. Besides early screening for risk detection, implications for prevention and intervention will depend on whether the association between ELS and IQ‐achievement discrepancy is causal, which we are not able to establish from the current study. If a causal role of ELS is supported by future research, this would suggest that primary prevention may help reduce underachievement. Furthermore, intervention strategies may help dampen the impact of ELS on IQ‐achievement discrepancy. The specific choice of intervention will however depend on which mechanisms link stress to IQ and academic achievement. On the one hand, high IQ might buffer the negative effects of ELS on academic achievement (i.e., IQ as moderator). Consistent with this idea, for example, high IQ has been proposed to increase resilience against the negative effects of adversity on academic achievement in a small study of adolescents (Masten et al., 1999). Under such a mechanism, intervention may be most effective when allocated primarily to children with lower levels of IQ, to help buffer the negative effects of ELS, as resources are then assigned to the most vulnerable group. Alternatively, ELS may increase risk for poor academic achievement by influencing IQ (i.e., IQ as mediator). Such a pathway is consistent with earlier literature showing that executive functioning mediates the negative effects of maternal psychopathology on academic achievement (Pearson et al., 2016). Under this scenario, intervention may focus more on enhancing coping skills and executive functioning in children exposed to ELS (Takacs & Kassai, 2019). Going forward, it will be important to disentangle the potential relation between these variables using advanced causal models.

The present study should be interpreted considering the following strengths and limitations. School achievement was measured with the national Cito test around age 12—an objective and standardized test that is widely used in Dutch primary schools to advise secondary school level (Van Boxtel et al., 2010). Furthermore, we utilized data from a large population‐based sample increasing the generalizability of our findings, and also included a wide range of stressors assessed prospectively from pregnancy to late childhood. As for limitations, a large number of children were excluded (i.e., 75.9% of our original sample) due to high frequencies of missing outcome data, which can bias results and limit generalizability of findings (Nohr et al., 2006). Nonetheless, sensitivity analyses using data additionally imputed for outcomes yielded largely consistent results. As for our outcome, we assessed IQ with a selection of subtests only. Nevertheless, the association between the shortened and the full version is high (Blok et al., 2022). Also, in our work, we focused on total IQ rather than verbal or performance IQ. As such, we cannot conclude if associations might differ for specific IQ types. In addition, a direct comparison between prenatal and postnatal ELS was not possible, as some of the risks assessed were only available or relevant at specific developmental periods (e.g., direct victimization of the child was only assessed postnatally). Besides, we could also not examine prenatal and postnatal ELS simultaneously in the same model, given the high correlation between them. Furthermore, when constructing the IQ‐achievement discrepancy, academic achievement at age 11.9 is predicted using IQ at age 13.6, meaning we predict backward in time. In the future, it would be more optimal to have both measures at the same time point, although, IQ is known to be stable over time (Schneider et al., 2014), so that results would not be expected to differ substantially. Lastly, the pathways linking ELS to the IQ‐achievement discrepancy remain unknown. It will be important to identify through which pathways ELS is associated with academic underachievement. For example, the IQ‐achievement discrepancy has been found to be associated with other sequelae of ELS, such as child psychiatric problems (Mayes et al., 2020) and problems with executive functioning (Alloway & Alloway, 2010; Ng & Hodges, 2020; Pechtel & Pizzagalli, 2011), which could pose as potential mechanisms linking ELS to academic underachievement.

In summary, in this prospective, population‐based study we identify ELS, measured both prenatally and postnatally, as a developmental risk factor for the IQ‐achievement discrepancy. Specifically, ELS beginning as early as in utero was found to be associated with lower academic achievement than would be expected based on IQ. Early life stressors often co‐occurred and showed high continuity over time, with effects primarily driven by shared variance across stress domains. Together, these findings point to ELS as an important prevention target in order to help children reach their full academic potential.

## Supporting information


Data S1

## References

[cdev13825-bib-0001] Ackerman, B. P. , Izard, C. E. , Schoff, K. , Youngstrom, E. A. , & Kogos, J. (1999). Contextual risk, caregiver emotionality, and the problem behaviors of six‐and seven‐year‐old children from economically disadvantaged families. Child Development, 70, 1415–1427. 10.1111/1467-8624.00103 10621964

[cdev13825-bib-0002] Alloway, T. P. , & Alloway, R. G. (2010). Investigating the predictive roles of working memory and IQ in academic attainment. Journal of Experimental Child Psychology, 106, 20–29. 10.1016/j.jecp.2009.11.003 20018296

[cdev13825-bib-0003] Appleyard, K. , Egeland, B. , van Dulmen, M. H. M. , & Sroufe, A. L. (2005). When more is not better: The role of cumulative risk in child behavior outcomes. Journal of Child Psychology and Psychiatry, 46, 235–245. 10.1111/j.1469-7610.2004.00351.x 15755300

[cdev13825-bib-0004] Benjamini, Y. , & Hochberg, Y. (1995). Controlling the false discovery rate: A practical and powerful approach to multiple testing. Journal of the Royal Statistical Society: Series B (Methodological), 57, 289–300. 10.1111/j.2517-6161.1995.tb02031.x

[cdev13825-bib-0047] Blok, E. , Schuurmans, I. K. , Tijburg, A. J. , Hillegers, M. , Koopman‐Verhoeff, M. E. , Muetzel, R. L. , Tiemeier, H. , & White, T. (2022). Cognitive performance in children and adolescents with psychopathology traits: a cross‐sectional multicohort study in the general population. Development and Psychopathology, 1–15. 10.1017/S0954579422000165 35249585

[cdev13825-bib-0005] Borghans, L. , Golsteyn, B. H. H. , Heckman, J. J. , & Humphries, J. E. (2016). What grades and achievement tests measure. Proceedings of the National Academy of Sciences, 113, 13354–13359. 10.1073/pnas.1601135113 PMC5127298 27830648

[cdev13825-bib-0006] Cecil, C. A. M. , Lysenko, L. J. , Jaffee, S. R. , Pingault, J.‐B. , Smith, R. G. , Relton, C. L. , Woodward, G. , McArdle, W. , Mill, J. , & Barker, E. D. (2014). Environmental risk, Oxytocin Receptor Gene (OXTR) methylation and youth callous‐unemotional traits: A 13‐year longitudinal study. Molecular Psychiatry, 19, 1071–1077. 10.1038/mp.2014.95 25199917 PMC4231290

[cdev13825-bib-0007] Cortes Hidalgo, A. P. , Neumann, A. , Bakermans‐Kranenburg, M. J. , Jaddoe, V. W. V. , Rijlaarsdam, J. , Verhulst, F. C. , White, T. , van IJzendoorn, M. H. , & Tiemeier, H. (2020). Prenatal maternal stress and child IQ. Child Development, 91, 347–365. 10.1111/cdev.13177 30376186

[cdev13825-bib-0008] de Maat, D. A. , Schuurmans, I. K. , Jongerling, J. , Metcalf, S. A. , Lucassen, N. , Franken, I. H. A. , Franken, I. H. A. , Prinzie, P. , & Jansen, P. W. (2021). Early life stress and behavior problems in early childhood: Investigating the contributions of child temperament and executive functions to resilience. Child Development, 93, e1–e16. 10.1111/cdev.13663 34448495 PMC9291511

[cdev13825-bib-0009] Derogatis, L. R. , & Melisaratos, N. (1983). The brief symptom inventory: An introductory report. Psychological Medicine, 13, 595–605. 10.1017/S0033291700048017 6622612

[cdev13825-bib-0010] Dipietro, J. A. , Costigan, K. A. , & Sipsma, H. L. (2008). Continuity in self‐report measures of maternal anxiety, stress, and depressive symptoms from pregnancy through two years postpartum. Journal of Psychosomatic Obstetrics & Gynecology, 29, 115–124. 10.1080/01674820701701546 18655259 PMC9566577

[cdev13825-bib-0011] Evans, G. W. (2004). The environment of childhood poverty. American Psychologist, 59, 77–92. 10.1037/0003-066X.59.2.77 14992634

[cdev13825-bib-0012] Gignac, G. E. , & Szodorai, E. T. (2016). Effect size guidelines for individual differences researchers. Personality and Individual Differences, 102, 74–78. 10.1016/j.paid.2016.06.069

[cdev13825-bib-0013] Hoffmann, J. P. (2020). Academic underachievement and delinquent behavior. Youth & Society, 52, 728–755. 10.1080/15374410903103502

[cdev13825-bib-0014] Horwood, J. , Salvi, G. , Thomas, K. , Duffy, L. , Gunnell, D. , Hollis, C. , Lewis, G. , Menezes, P. , Thompson, A. , Wolke, D. , Zammit, S. , & Harrison, G. (2008). IQ and non‐clinical psychotic symptoms in 12‐year‐olds: Results from the ALSPAC birth cohort. The British Journal of Psychiatry, 193, 185–191. 10.1192/bjp.bp.108.051904 18757973 PMC2806573

[cdev13825-bib-0015] Iacobucci, D. (2010). Structural equations modeling: Fit indices, sample size, and advanced topics. Journal of Consumer Psychology, 20, 90–98. 10.1016/j.jcps.2009.09.003

[cdev13825-bib-0016] Kaya, F. , Stough, L. M. , & Juntune, J. (2016). The effect of poverty on the verbal scores of gifted students. Educational Studies, 42, 85–97. 10.1080/03055698.2016.1148585

[cdev13825-bib-0017] Kingston, D. , Tough, S. , & Whitfield, H. (2012). Prenatal and postpartum maternal psychological distress and infant development: A systematic review. Child Psychiatry & Human Development, 43, 683–714. 10.1007/s10578-012-0291-4 22407278

[cdev13825-bib-0046] Kooijman, M. N. , Kruithof, C. J. , van Duijn, C. M. , Duijts, L. , Franco, O. H. , van IJzendoorn, M. H. , Jongst, J. C. , Klaver, C. C. W. , van der Lugt, A. , Mackenbach, J. P. , Moll, H. A. , Peeters, R. P. , Raat, H. , Rings, E. H. H. M. , Rivadeneira, F. , van der Schroeff, M. P. , Steegers, E. A. P. , Tiemeier, H. , Uitterlinden, A. G. , Verhulst, F. C. , Wolvius, E. , Felix, J. F. , & Jaddoe, V. W. (2016). The Generation R Study: Design and cohort update 2017. European Journal of Epidemiology, 31(12), 1243–1264. 10.1007/s10654-016-0224-9 28070760 PMC5233749

[cdev13825-bib-0018] Lau, K.‐L. , & Chan, D. W. (2001). Identification of underachievers in Hong Kong: Do different methods select different underachievers? Educational Studies, 27, 187–200. 10.1080/03055690120050419

[cdev13825-bib-0019] Lesuis, S. L. , Hoeijmakers, L. , Korosi, A. , de Rooij, S. R. , Swaab, D. F. , Kessels, H. W. , Lucassen, P. J. , & Krugers, H. J. (2018). Vulnerability and resilience to Alzheimer's disease: Early life conditions modulate neuropathology and determine cognitive reserve. Alzheimer's Research & Therapy, 10, 1–20. 10.1186/s13195-018-0422-7 PMC6145191 30227888

[cdev13825-bib-0020] Liming, K. W. , & Grube, W. A. (2018). Wellbeing outcomes for children exposed to multiple adverse experiences in early childhood: A systematic review. Child and Adolescent Social Work Journal, 35, 317–335. 10.1007/s10560-018-0532-x

[cdev13825-bib-0021] Lupien, S. J. , McEwen, B. S. , Gunnar, M. R. , & Heim, C. (2009). Effects of stress throughout the lifespan on the brain, behaviour and cognition. Nature Reviews Neuroscience, 10, 434–445. 10.1038/nrn2639 19401723

[cdev13825-bib-0022] Masten, A. S. , Hubbard, J. J. , Gest, S. D. , Tellegen, A. , Garmezy, N. , & Ramirez, M. (1999). Competence in the context of adversity: Pathways to resilience and maladaptation from childhood to late adolescence. Development and Psychopathology, 11, 143–169. 10.1017/S0954579499001996 10208360

[cdev13825-bib-0023] Mayes, S. D. , Waschbusch, D. A. , Calhoun, S. L. , & Mattison, R. E. (2020). How common are academic overachievement and underachievement in children with autism or ADHD? Journal of Developmental and Physical Disabilities, 32, 775–783. 10.1007/s10882-019-09719-8

[cdev13825-bib-0024] McCall, R. B. , Evahn, C. , & Kratzer, L. (1992). High school underachievers: What do they achieve as adults? (Vol. 1). Sage Publications, Inc.

[cdev13825-bib-0025] Morales, J. R. , & Guerra, N. G. (2006). Effects of multiple context and cumulative stress on urban children's adjustment in elementary school. Child Development, 77, 907–923. 10.1111/j.1467-8624.2006.00910.x 16942497

[cdev13825-bib-0026] Najman, J. M. , Hayatbakhsh, M. R. , Clavarino, A. , Bor, W. , O'Callaghan, M. J. , & Williams, G. M. (2010). Family poverty over the early life course and recurrent adolescent and young adult anxiety and depression: A longitudinal study. American Journal of Public Health, 100, 1719–1723. 10.2105/AJPH.2009.180943 20634459 PMC2920957

[cdev13825-bib-0027] Ng, R. , & Hodges, E. K. (2020). Associations between attention regulation, working memory, and academic skills among pediatric patients with epilepsy. Advances in Neurodevelopmental Disorders, 4, 59–66. 10.1007/s41252-019-00137-7

[cdev13825-bib-0028] Nohr, E. A. , Frydenberg, M. , Henriksen, T. B. , & Olsen, J. (2006). Does low participation in cohort studies induce bias? Epidemiology, 17, 413–418. 16755269 10.1097/01.ede.0000220549.14177.60

[cdev13825-bib-0029] Pearson, R. M. , Bornstein, M. H. , Cordero, M. , Scerif, G. , Mahedy, L. , Evans, J. , Abioye, A. , & Stein, A. (2016). Maternal perinatal mental health and offspring academic achievement at age 16: The mediating role of childhood executive function. Journal of Child Psychology and Psychiatry, 57, 491–501. 26616637 10.1111/jcpp.12483 PMC4789117

[cdev13825-bib-0030] Pechtel, P. , & Pizzagalli, D. A. (2011). Effects of early life stress on cognitive and affective function: An integrated review of human literature. Psychopharmacology, 214, 55–70. 10.1007/s00213-010-2009-2 20865251 PMC3050094

[cdev13825-bib-0031] R Core Team . (2020). R: A language and environment for statistical computing. (Version 3.6.3.). R Foundation for Statistical Computing.

[cdev13825-bib-0032] Reis, S. M. , & McCoach, D. B. (2000). The underachievement of gifted students: What do we know and where do we go? Gifted child quarterly, 44, 152–170. 10.1177/001698620004400302

[cdev13825-bib-0033] Rosseel, Y. (2012). Lavaan: An R package for structural equation modeling and more. Version 0.5–12 (BETA). Journal of Statistical Software, 48, 1–36. 10.18637/jss.v048.i02

[cdev13825-bib-0034] Schneider, W. , Niklas, F. , & Schmiedeler, S. (2014). Intellectual development from early childhood to early adulthood: The impact of early IQ differences on stability and change over time. Learning and Individual Differences, 32, 156–162. 10.1016/j.lindif.2014.02.001

[cdev13825-bib-0035] Statistics Netherlands . (2004). Immigrants in the Netherlands 2004 (Allochtonen in Nederland 2004). Statistics Netherlands (Centraal Bureau voor de Statistiek).

[cdev13825-bib-0048] Steenkamp, L. R. , Bolhuis, K. , Blanken, L. M. , Luijk, M. P. , Hillegers, M. H. , Kushner, S. A. , & Tiemeier, H. (2021). Psychotic experiences and future school performance in childhood: A population‐based cohort study. Journal of Child Psychology and Psychiatry, 62(3), 357–365. 32559319 10.1111/jcpp.13281 PMC7983885

[cdev13825-bib-0036] Stern, Y. , Arenaza‐Urquijo, E. M. , Bartrés‐Faz, D. , Belleville, S. , Cantilon, M. , Chetelat, G. , Ewers, M. , Franzmeier, N. , Kempermann, G. , Kremen, W. S. , Okonkwo, O. , Scarmeas, N. , Soldan, A. , Udeh‐Momoh, C. , Valenzuela, M. , Vemuri, P. , & Vuoksimaa, E. (2020). Whitepaper: Defining and investigating cognitive reserve, brain reserve, and brain maintenance. Alzheimer's & Dementia, 16, 1305–1311. 10.1016/j.jalz.2018.07.219 PMC6417987 30222945

[cdev13825-bib-0037] Takacs, Z. K. , & Kassai, R. (2019). The efficacy of different interventions to foster children's executive function skills: A series of meta‐analyses. Psychological Bulletin, 145, 653–697. 31033315 10.1037/bul0000195

[cdev13825-bib-0038] Van Boxtel, H. , Engelen, R. , & De Wijs, A. (2010). Wetenschappelijke verantwoording van de Eindtoets Basisonderwijs 2010 [Scientific justification of the Final Test Primary Education 2010]. Cito.

[cdev13825-bib-0039] Van Buuren, S. (2018). Flexible imputation of missing data (2nd ed.). Chapman and Hall/CRC.

[cdev13825-bib-0040] Van Buuren, S. , & Groothuis‐Oudshoorn, K. (2011). mice: Multivariate imputation by chained equations in R. Journal of Statistical Software, 45, 1–67. 10.3978/j.issn.2305-5839.2015.12.63

[cdev13825-bib-0041] Van der Lubbe, M. (2007). The end of primary school test (better known as Citotest) . Paper presented at the 33rd annual conference of the International Association for Educational Assessment.

[cdev13825-bib-0042] Vatcheva, K. P. , Lee, M. , McCormick, J. B. , & Rahbar, M. H. (2016). Multicollinearity in regression analyses conducted in epidemiologic studies. Epidemiology: Open Access, 6, 1–9. 10.4172/2161-1165.1000227 PMC4888898 27274911

[cdev13825-bib-0043] Vogel, S. C. , Perry, R. E. , Brandes‐Aitken, A. , Braren, S. , & Blair, C. (2021). Deprivation and threat as developmental mediators in the relation between early life socioeconomic status and executive functioning outcomes in early childhood. Developmental Cognitive Neuroscience, 47, 1–10. 10.1016/j.dcn.2020.100907 PMC7777490 33383555

[cdev13825-bib-0044] Wechsler, D. (2014). WISC‐V: Technical and interpretive manual. NCS Pearson, Incorporated.

[cdev13825-bib-0045] Young‐Southward, G. , Eaton, C. , O'Connor, R. , & Minnis, H. (2020). Investigating the causal relationship between maltreatment and cognition in children: A systematic review. Child Abuse & Neglect, 107, 1–17. 10.1016/j.chiabu.2020.104603 32599461

